# Curcumin Promotes KLF5 Proteasome Degradation through Downregulating YAP/TAZ in Bladder Cancer Cells

**DOI:** 10.3390/ijms150915173

**Published:** 2014-08-28

**Authors:** Yang Gao, Qi Shi, Shan Xu, Chong Du, Liang Liang, Kaijie Wu, Ke Wang, Xinyang Wang, Luke S. Chang, Dalin He, Peng Guo

**Affiliations:** 1 Department of Urology, the First Affiliated Hospital of Xi’an Jiaotong University, Xi’an 710061, China; E-Mails: gaoyangxjtu@126.com (Y.G.); sq_880702@163.com (Q.S.); shanhuxs@163.com (S.X.); 125302400@163.com (C.D.); panda1773@gmail.com (L.L.); yidaiwujin@126.com (Ka.W.); shishan_86@163.com (Ke.W.); wangxinyang0929@163.com (X.W.); changlukes@yahoo.com (L.S.C.); dalinhexjtu@163.com (D.H.); 2 Oncology Research Lab, Key Laboratory of Environment and Genes Related to Diseases, Ministry of Education, Xi’an 710061, China

**Keywords:** KLF5, curcumin, bladder cancer, protein degradation, YAP, TAZ

## Abstract

KLF5 (Krüppel-like factor 5) plays critical roles in normal and cancer cell proliferation through modulating cell cycle progression. In this study, we demonstrated that curcumin targeted KLF5 by promoting its proteasome degradation, but not by inhibiting its transcription in bladder cancer cells. We also demonstrated that lentivirus-based knockdown of KLF5 inhibited cancer cell growth, while over-expression of a Flag-tagged KLF5 could partially reverse the effects of curcumin on cell growth and cyclin D1 expression. Furthermore, we found that curcumin could down-regulate the expression of Hippo pathway effectors, YAP and TAZ, which have been reported to protect KLF5 protein from degradation. Indeed, knockdown of YAP by small interfering RNA caused the attenuation of KLF5 protein, but not KLF5 mRNA, which was reversed by co-incubation with proteasome inhibitor. A xenograft assay in nude mice finally proved the potent inhibitory effects of curcumin on tumor growth and the pro-proliferative YAP/TAZ/KLF5/cyclin D1 axis. Thus, our data indicates that curcumin promotes KLF5 proteasome-dependent degradation through targeting YAP/TAZ in bladder cancer cells and also suggests the therapeutic potential of curcumin in the treatment of bladder cancer.

## 1. Introduction

In 2014, 74,690 new bladder cancer cases and 15,580 cancer deaths are estimated to occur in the United States. Among men, it is the fourth most common cancer and is the eighth leading cause of cancer death [1]. Generally, non-muscle invasive superficial bladder cancers are treated by transurethral resection following intravesical chemotherapy or immunotherapy, but the recurrence rate is still high, and some cases progress to a higher grade [2]. Thus, efforts to uncover the molecular mechanisms of bladder cancer progression and to develop novel agents to target relevant molecules or pathways can help patients to achieve better therapeutic efficacy.

KLF5 (Krüppel-like factor 5), a member of the Krüppel-like factor (KLF) family, has been shown to play important roles in the development of several types of human cancers by modulating the transcription of its target genes [3,4]. Deletion of *KLF5* from the developing bladder urothelium blocked epithelial cell differentiation and impaired bladder morphogenesis and function in mice [5]. Moreover, exogenous KLF5 expression increased cell cycle transition and up-regulated cyclin D1 in TSU-Pr1 human bladder cancer cells [6]. These findings suggest a pro-oncogenic role of KLF5 in bladder cancer. On the other hand, post-transcriptional modifications, especially ubiquitination of KLF5 protein, can greatly affect its functional display. Several E3 ubiquitin ligases, including WWP1, FBW7 and SMURF2, promote ubiquitination and degradation of KLF5 [7,8,9]. Additionally, YAP and TAZ, two effectors of the Hippo tumor suppressor pathway, can inhibit WWP1–KLF5 protein interaction and stabilize KLF5 [10,11]. Therefore, as an important growth-promoting gene, *KLF5* could be a candidate target for bladder cancer treatment, and modulating its degradation will be an efficient approach to inhibit KLF5.

Curcumin, a hydrophobic polyphenol derived from turmeric (*Curcuma longa*), is one of the most studied plant-derived natural products. In various human cancers, curcumin has shown anti-proliferation, apoptosis induction, chemoprevention/sensitivity, anti-angiogenesis and anti-invasion/metastasis properties [12,13,14]. In bladder cancer, curcumin also has shown a promising anticancer activity [15,16]. Although inhibition of NF-κB-dependent genes is one of the most predominant effects of curcumin, its potential targets have been expanded to a wide range of other pathways and molecules [12]. KLF5 has been shown to play critical roles in proliferation and tumorigenesis in several cancer types, including bladder cancer [3,6]. Therefore, we proposed a hypothesis that KLF5 could be an important target of curcumin in bladder cancer cells.

In the present study, using *in vitro* and *in vivo* assays, we determined whether KLF5 was a target of curcumin and whether KLF5 played a role in the anti-proliferative function of curcumin. Mechanistically, we further investigated the effects of curcumin on the expression of KLF5-related E3 ubiquitin ligases and YAP/TAZ. We also examined whether KLF5 expression was affected by YAP knockdown. Moreover, we determined whether curcumin inhibited the growth of bladder cancer in a xenograft mouse model.

## 2. Results

### 2.1. Curcumin Down-Regulated KLF5 Protein Expression in a Dose- and Time-Dependent Manner in 5637 and WH Bladder Cancer Cells

**Figure 1:**
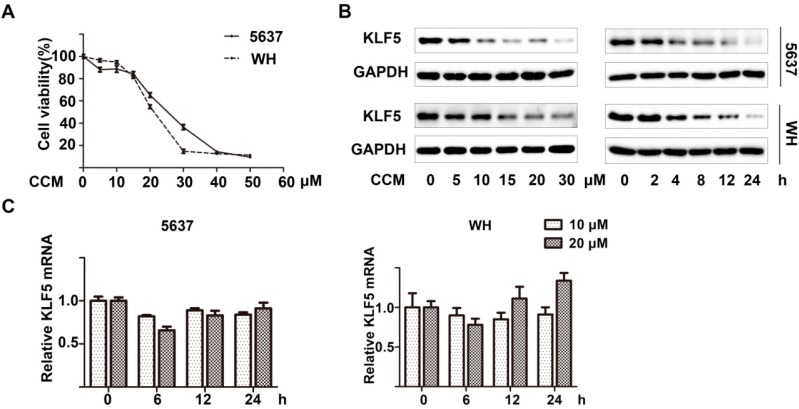
Curcumin down-regulated KLF5 protein expression in a dose- and time-dependent manner. (**A**) 5637 and WH bladder cancer cells were treated with the indicated concentration of curcumin (CCM) for 48 h; then the cell viability was determined by the 3-(4,5-dimethylthiazol-2-yl)-2,5-diphenyltetrazolium bromide (MTT) assay; (**B**) After being treated with various concentrations of curcumin for 12 h or with 15 μM of curcumin for the indicated period, KLF5 protein expression was determined by western blot analysis;and (**C**) KLF5 mRNA expression levels were analyzed by real-time qPCR after 5637 and WH cells were treated with 10 or 20 μM curcumin for 6, 12 and 24 h. Results were presented as mean ± SD from three independent experiments.

Curcumin inhibited the cell viability of 5637 and WH human bladder cancer cells in a dose-dependent manner after 48 h of treatment, as determined by the 3-(4,5-dimethylthiazol-2-yl)-2,5-diphenyltetrazolium bromide (MTT) assay (Figure 1A). Through western blot analysis, we also found that KLF5 protein expression decreased with increasing curcumin concentration (0–30 μM) or prolonging treatment (0–24 h) in both cell lines (Figure 1B). To further determine whether the transcription inhibition of KLF5 was involved, we performed a real-time qPCR assay to analysis KLF5 mRNA expression and found that along with the curcumin treatment, the mRNA level of KLF5 was not decreased significantly, which was not consistent with the protein level decrease (Figure 1C). These results indicated that curcumin could decrease KLF5 protein expression via a post-transcriptional regulation.

### 2.2. Curcumin Promoted Proteasome-Dependent Degradation of KLF5 Protein

**Figure 2:**
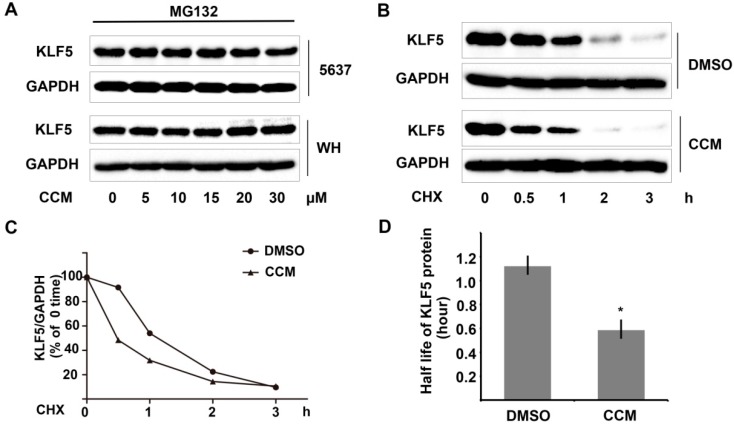
Curcumin promoted proteasome-dependent degradation of KLF5 protein. (**A**) 5637 and WH cells were pretreated with 10 μM MG132 for one hour before the treatment of curcumin. Twelve hours later, the whole-cell lysates were harvested, and KLF5 protein was analyzed by western blot; (**B**) KLF5 protein attenuation in the cycloheximide (CHX) chase assay was examined after DMSO or curcumin treatment in 5637 cells; (**C**) KLF5 protein levels were quantified using Image Lab software and were normalized to GAPDH; and (**D**) KLF5 half-life values were calculated by nonlinear regression analysis with GraphPad Prism software. The results represent three independent experiments with similar results. Error bars show the 95% confidence intervals. * *p* < 0.05.

We further investigated whether the protein stability of KLF5 was decreased by curcumin. Indeed, pretreating 5637 cells with proteasome inhibitor MG132 abolished the down-regulation of KLF5 protein after curcumin treatment (Figure 2A), which suggested that curcumin promotes proteasome-dependent degradation of KLF5. Next, we used a cycloheximide (CHX) chase assay to examine whether the half-life of KLF5 protein was affected by curcumin treatment. Unlike the DMSO control group, curcumin pretreatment accelerated KLF5 protein degradation in the presence of CHX (Figure 2B). After being normalized to GAPDH, the results were plotted as the relative KLF5 levels compared with those at the zero time of CHX treatment (Figure 2C). The half-life value of KLF5 was calculated by nonlinear regression analysis using GraphPad Prism software (GraphPad, San Diego, CA, USA). The putative half-life of KLF5 decreased from 1.121 h (95% confidence interval (CI), 0.942 to 1.384) to 0.585 (95% CI, 0.521 to 0.667) (Figure 2D). Therefore, our data demonstrated that curcumin could promote proteasome-dependent degradation of KLF5 protein.

### 2.3. KLF5 Mediated the Anti-Proliferative Effect of Curcumin

**Figure 3:**
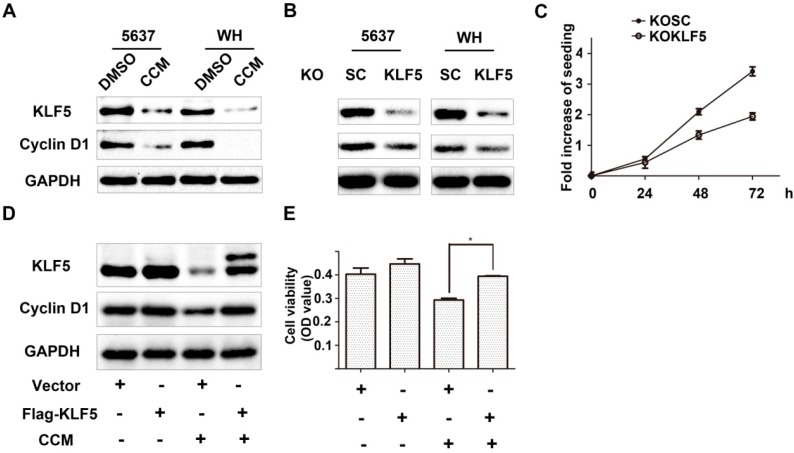
KLF5 mediated the anti-proliferative effect of curcumin. (**A**,**B**) KLF5 and cyclin D1 expression in curcumin-treated 5637 and WH cells (**A**) and in lentivirus-shKLF5-infected stable cells (**B**), as detected by western blot analysis; (**C**) The growth rates of 5637 KOSC and KOKLF5 cells were determined by the MTT assay; (**D**) Flag-KLF5 or vector-transfected 5637 cells were treated with 15 μM curcumin for 24 h. Then, cell lysates were harvested, and the KLF5 and cyclin D1 levels were analyzed by the western blot assay;and (**E**) The cell viability of different groups in (**D**) was determined by the MTT assay. The results represent three independent experiments. The values are the mean ± SD. * *p* < 0.05.

To further clarify whether accelerated degradation of KLF5 might play roles in the anti-proliferative effect of curcumin, we modified the expression level of KLF5 and determined the changes of its well-known target gene *cyclin D1* expression and cell growth. Firstly, our results showed that both curcumin treatment and lentivirus-shKLF5 inhibited the expression of cyclin D1 protein (Figure 3A,B). Moreover, stable established KLF5 knockdown 5637/KOKLF5 cells consistently displayed a reduced growth rate compared with scramble control 5637/KOSC cells (Figure 3C). Because the KLF5 with Flag tagged at the *N*-terminal is more stable than KLF5 alone [17], we transfected 5637 cells with a Flag-tagged KLF5-expressing plasmid and its control vector. KLF5 and cyclin D1 expression in the Flag-KLF5 group were not higher than the control group in the normal condition. However, after curcumin treatment, which rapidly decreased endogenous KLF5 protein, the appearance of Flag-KLF5 induced a higher level of cyclin D1 (Figure 3D). Consistently, Flag-KLF5-transfected cells also showed higher cell viability compared with the control cells after treatment with 15 μΜ curcumin for 24 h (Figure 3E). These results indicated that a more stable form of Flag-KLF5 protein could partially reverse curcumin-induced growth arrest and cyclin D1 suppression. Therefore, downregulation of KLF5 may be essential for curcumin to inhibit bladder cancer cell growth.

### 2.4. Curcumin Down-Regulated YAP and TAZ Expression

**Figure 4:**
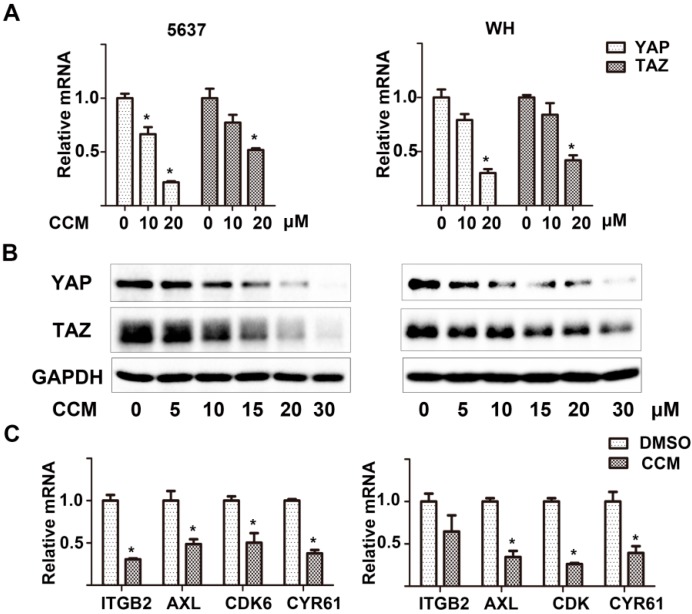
Curcumin targeted YAP and TAZ. (**A**) The mRNA levels of YAP and TAZ in 5637 (**left**) and WH (**right**) cells treated with 0, 10 or 20 μM curcumin for 12 h as determined by real-time qPCR; (**B**) After treatment with curcumin for 12 h, YAP and TAZ protein levels were determined by western blot analysis in the two cells; (**C**) The mRNA level of *YAP/TAZ* downstream genes in 5637 and WH cells after treatment with 20 μM curcumin for 24 h, as detected by real-time qPCR. Results were presented as the mean ± SD from three independent experiments. * *p* < 0.05.

Mechanistically, we assumed that curcumin might regulate the expression of E3 ubiquitin ligases and then mediate KLF5 ubiquitination and degradation, but real-time qPCR assays showed that there was no significant changes of several well-identified KLF5 targeting E3 ubiquitin ligases, including WW domain containing E3 ubiquitin protein ligase 1 (WWP1), F-box and WD repeat domain containing 7 (FBW7) and SMAD specific E3 ubiquitin protein ligase 2 (SMURF2) (Supplementary Figure S1). Interestingly, both mRNA and protein levels of two Hippo pathway effectors, YAP and TAZ, which had been reported to competitively antagonize WWP1’s binding with KLF5, were significantly down-regulated by curcumin in a dose-dependent manner (Figure 4A,B). Furthermore, the downstream targets of YAP and TAZ, including integrin, beta 2 (ITGB2), AXL receptor tyrosine kinase (AXL), cyclin-dependent kinase 6 (CDK6) and cysteine-rich, angiogenic inducer, 61 (CYR61), were also down-regulated after curcumin treatment (Figure 4C). These results revealed that curcumin decreased *YAP* and *TAZ* gene expression, which could lead to the decease of KLF5 protein stability.

### 2.5. YAP Played Critical Roles in KLF5 Protein Stability in 5637 Cells

**Figure 5:**
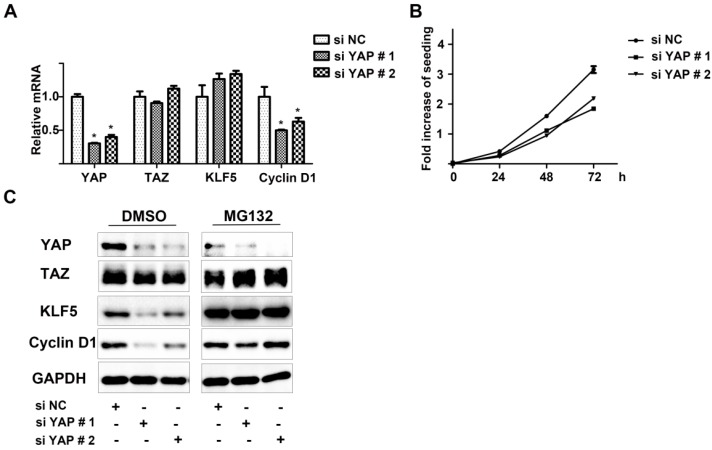
Knockdown of YAP attenuated KLF5 protein, but not mRNA expression. (**A**) 5637 cells were transfected with siRNAs specifically targeting YAP (si YAP # 1,2) or non-specific control (si NC). Thirty six hours after transfection, total RNAs were isolated, and the expression of YAP, TAZ, KLF5 and cyclin D1 were analyzed by real-time qPCR; (**B**) Twelve hours after transfection, cells were trypsinized and seeded for an MTT assay; (**C**) After DMSO or MG132 (10 μM) treatment for 12 h, siRNA-transfected cells were lysed for western blot analysis by using antibodies specific to YAP, TAZ, KLF5 and cyclin D1. The data shown are representative of three independent experiments with similar results; bars, SDs. * *p* < 0.05.

The important roles of YAP and TAZ in maintaining KLF5 protein stability have been shown in other systems, but whether the same mechanism exists in bladder cancer remains to be determined. In 5637 cells, we used small interfering RNA (siRNA) to knockdown YAP and then to study its effects on KLF5 and cyclin D1 expression, as well as cell proliferation. After transfection, two differently-designed YAP-specific siRNAs effectively impaired YAP mRNA compared with the negative control group (Figure 5A). The expression of TAZ and KLF5 mRNAs was not affected, which not only showed the specificity of the designed siRNAs, but also meant that transcriptional suppression was not involved in YAP-induced KLF5 protein attenuation (Figure 5C, DMSO group). However, treatment with proteasome inhibitor MG132 rescued the KLF5 protein instability caused by YAP down-regulation (Figure 5C, MG132 group). Consistent with KLF5 expression, cyclin D1 expression at both the mRNA and protein level was also affected by YAP knockdown (Figure 5A,C). The proliferation rates of 5637 bladder cancer cells were decreased after transfection, as determined by the MTT assay (Figure 5B). These data demonstrated the important role of YAP in maintaining KLF5 protein stability and also suggested that the pro-proliferative YAP/TAZ/KLF5/cyclin D1 axis might be an attractive target in bladder cancer.

### 2.6. Curcumin Inhibited Subcutaneous Tumor Growth and the YAP/TAZ/KLF5/Cyclin D1 Axis in Vivo

**Figure 6:**
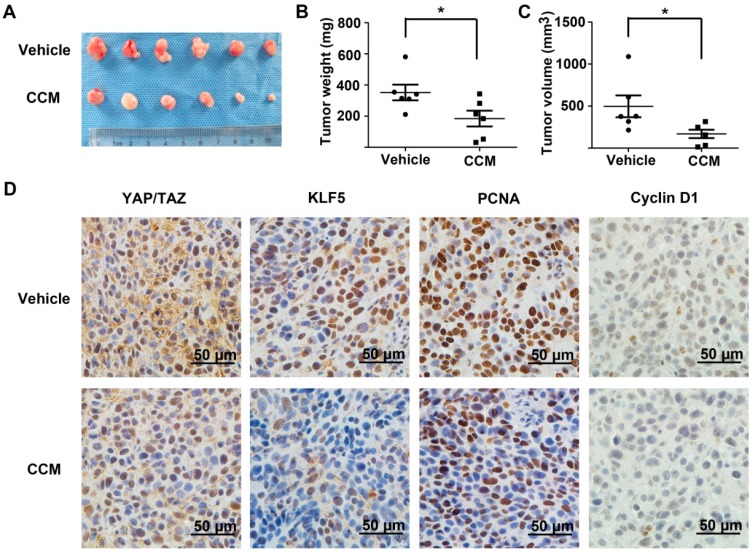
Antitumor effects of curcumin on 5637 xenografts in nude miceand the YAP/TAZ/KLF5/cyclin D1 axis *in vivo*. (**A**) Tumors from 5637 implanted nude mice, treated with vehicle or 200 mg/kg/day curcumin for three weeks; Tumor weight (**B**) and volume (**C**) were compared between the two groups; and (**D**) Representative photograph (400×) of immunohistochemistry (IHC) staining of YAP/TAZ, KLF5, PCNA and cyclin D1 expression in tumor sections from the vehicle and curcumin-treated xenografts. * *p* < 0.05.

Next, we want to confirm the inhibitory effects of curcumin on tumor growth and the YAP/KLF5/cyclin D1 axis in a nude mice xenograft model. 5637 bladder cancer cells (1 × 10^6^) were transplanted into the right flank of nude mice. Seven days later, vehicle or curcumin was injected intraperitoneally for the next three weeks. Compared with the vehicle group, curcumin inhibited tumor growth at the end of the assay (Figure 6A). Both the weight and volume of the curcumin-treated group tumors were significantly inhibited by curcumin (Figure 6B,C). Consistently, proliferating cell nuclear antigen (PCNA) staining in xenograft tissues was also evidently inhibited after curcumin treatment (Figure 6D). Importantly, curcumin treatment also caused potent suppressive effects on YAP/TAZ, KLF5 and cyclin D1 expression, which was consistent with our *in vitro* results. These results further supported that curcumin suppress tumor growth and the YAP/TAZ/KLF5/cyclin D1 axis in bladder cancer in a xenograft tumor nude mouse model.

## 3. Discussion

Although KLF5 inhibits cell growth or promotes cell apoptosis in prostate cancer and esophageal squamous cell cancer [18,19], it has been reported that KLF5 plays an oncogenic role in other cancers. For example, KLF5 has been identified as a therapeutic target in colorectal cancer, and a small molecule, compound CID 5951923, which specifically inhibits KLF5 expression, has been developed through high throughput screening [20]. Exogenous KLF5 promotes TSU-pr1 bladder cancer cell growth *in vitro* and *in vivo*, which is consistent with our results that knockdown of KLF5 leads to the down-regulation of cyclin D1 and a decrease of the cell proliferation of 5637 bladder cancer cells (Figure 3). These results suggest that KLF5 could also be a target in the treatment of bladder cancer.

Like other crucial transcription factors, such as p53 and c-MYC, KLF5 is rapidly metabolized. The KLF5 protein half-life is about 1.5 h by pulse chase assays [3,4]. In the present study, we found that the expression level of KLF5 protein, but not KLF5 mRNA, was significantly attenuated by curcumin, indicating a post-transcriptional regulation mechanism behind. In 5637 and WH cells pretreated with proteasome inhibitor MG132, the down-regulation of KLF5 protein by curcumin was inhibited; moreover, through the cycloheximide chase assay, we found that the half-life of KLF5 protein in 5637 cells was significantly decreased after curcumin treatment (Figure 2). These data demonstrated that KLF5 was a new candidate target for curcumin and that curcumin might activate the proteasome pathway to impair KLF5 protein stability. Since KLF5 was identified as an oncogenic molecule in intestinal cancer and estrogen receptor (ER)-negative breast cancers [4], curcumin or its derivatives may also be applied to these cancers to manipulate the KLF5 levels.

In this study, we found that curcumin decreased the proliferation of both 5637 and WH bladder cancer cells, and KLF5 was down-regulated by curcumin (Figure 1). Furthermore, by transfecting KLF5 shRNA or a stable KLF5, which was fused with a Flag tag on its *N*-terminal [17], we found that the KLF5 could antagonize the inhibitory effect of curcumin on cell growth (Figure 3D,E). This demonstrated that down-regulation of KLF5 might be essential for curcumin to inhibit bladder cancer cell growth.

Cyclin D1, a key regulator of cell cycle progression, is frequently amplified and over-expressed in cancers [21]. Our results showed that curcumin inhibited the expression of cyclin D1 in both 5637 and WH cell lines, which is consistent with previous reports [22]. Cyclin D1 is a typical downstream target of KLF5 [6,23]. Curcumin treatment or knockdown of KLF5 by lentivirus-shKLF5 down-regulated cyclin D1 expression and decreased cancer cell growth. Moreover, over-expression of Flag-tagged KLF5 partially reversed curcumin-induced cyclin D1 down-regulation and growth inhibition in 5637 cells. These results indicated a potential role of KLF5 in curcumin-induced suppression of bladder cancer proliferation.

The decrease in the level of numerous proteins (*i.e*., Sp1, ErbB2 and cyclin E1) after curcumin treatment has been reported [16,24,25], but the mechanism has not been well elucidated. Previous studies have shown that curcumin directly inhibited proteasome activity *in vitro* and *in vivo* [26,27], which resulted in the accumulation of certain ubiquitinated proteins, such as IκB-α and Bax. In this study, we made efforts to uncover the mechanism behind the accelerated KLF5 protein attenuation caused by curcumin. The mRNA level of WWP1, FBW7 and SMURF2, which have been reported to interact with KLF5 and mediate the ubiquitination of KLF5, were not significantly up-regulated by curcumin in 5637 and WH bladder cancer cells (Supplementary Figure S1). On the other hand, because KLF5 protein stability can also be increased by Hippo pathway effector YAP/TAZ [10,11], we turned to investigate whether curcumin regulates KLF5 through YAP/TAZ modulation. Both the real-time qPCR assay and western blot analysis results indicated that YAP/TAZ was downregulated by the treatment of curcumin (Figure 4A,B). Furthermore, several downstream targets of YAP/TAZ were also down-regulated after curcumin treatment (Figure 4C). Our results confirmed that YAP/TAZ is down-regulated by curcumin, and because they stabilize KLF5 in cancer cells, the decrease of YAP/TAZ may lead to the degradation of KLF5. Although the requirement of YAP and TAZ in maintaining KLF5 protein stability has been reported in breast cancer [10,11], whether the same mechanism functions in bladder cancer remains to be proven. Two different siRNAs designed to target YAP not only knocked-down YAP effectively, but also suppressed 5637 cells’ growth rate, down-regulated KLF5 protein (not mRNA) and its target gene cyclin D1. What is more, proteasome inhibitor MG132 could reversed this KLF5 protein attenuation (Figure 5). Thus, our study connected YAP and KLF5 in bladder cancer, and the pro-proliferative YAP/TAZ/KLF5/cyclin D1 axis was also revealed. Importantly, the *in vivo* study further confirmed the potent inhibitory effects of curcumin on tumor growth and this axis (Figure 6). In conclusion, we found that KLF5 promotes the growth of bladder cancer cells and that curcumin suppress the KLF5 protein level in a proteasome-dependent way. Mechanistically, YAP is essential for the stability of KLF5, and curcumin inhibits KLF5 at least partially through suppressing the expression of YAP/TAZ. Thus, the YAP/TAZ/KLF5/cyclinD1 axis is important for the growth of bladder cancer, and it is a potential therapeutic target for curcumin and other chemicals in cancer treatment.

## 4. Experimental Section

### 4.1. Cell Culture and Reagents

Human bladder cancer 5637 and WH cells were cultured in RPMI-1640 (5637) or DMEM (WH) medium supplemented with 10% fetal bovine serum at 37 °C, aired with 5% CO_2_.

Curcumin and proteasome inhibitor MG132 were obtained from Sigma–Aldrich (St. Louis, MO, USA). Polybrene and the protein synthesis inhibitor, CHX, were purchased from Biyuntian (Shanghai, China). These reagents were dissolved in DMSO and stored at −20 °C. The final concentration of DMSO for all treatments (including controls) was maintained at less than 0.5%.

### 4.2. 3-(4,5-Dimethylthiazol-2-yl)-2,5-diphenyltetrazolium bromide (MTT) Assay

Cell viability and growth rate were measured by the3-(4,5-dimethylthiazol-2-yl)-2,5-diphenyltetrazolium bromide (MTT) assay, as described previously [28]. Briefly, 5 × 10^3^ normal cultured or transfected cells were seeded in 96-well culture plates, followed by various treatments for the indicated time, and then, cells were washed once and incubated with 0.5 mg/mL of MTT at 37 °C for 4 h. The medium was discarded carefully and 150 μL DMSO were added to solubilize the formazan crystals. Finally, the absorbance was measured for each well at a wavelength of 490 nm using the Microplate Autoreader (Bio-Tek Instruments Inc., Winooski, VT, USA). Independent experiments were repeated in triplicate.

### 4.3. Western Blot Analysis

At the end of treatment, cultured cells were washed once with cold PBS and lysed in RIPA buffer (50 mM Tris pH 8.0, 150 mM NaCl, 0.1% SDS, 1% NP-40 and 0.5% sodium deoxycholate) containing protease inhibitors. Approximately 30 μg of protein were separated with 10%–12% SDS-PAGE gel and blotted onto nitrocellulose membranes. The membranes were blocked with 5% skim milk at room temperature for 1 h and then incubated with primary antibodies against GAPDH (1:10,000, Shanghai Kangchen, Shanghai, China), KLF5 (previously described [29], 1:500) or YAP/TAZ (#8418, 1:800, Cell Signaling, Beverly, MA, USA) at 4 °C overnight, followed by a TBST wash and 1 h incubation with horseradish peroxidase-conjugated secondary antibodies at room temperature. Protein bands were visualized by a Molecular Imager ChemiDoc XRS System (Bio-Rad Laboratories, Hercules, CA, USA).

### 4.4. Cycloheximide (CHX) Chase Assay

5637 cells were treated with DMSO or 15 μM curcumin for 2 h, then washed with PBS 3 times and refed with medium containing 10 μg/mL CHX. The whole-cell lysates were harvested at the indicated time and subsequently subjected to a western blot analysis.

### 4.5. Plasmids, Lentivirus Preparation, siRNA and Transfection

PLKO.1 lentiviral vectors encoding short hairpin RNA (shRNA) targeting human KLF5 or scramble shRNA (SC), as the control, were constructed by GenPharma (Shanghai, China). The target sequence of the selected shRNA is sh710: GGTTACCTTACAGTATCAACA. To generate lentivirus, PAX2, VSV-G and the plasmids described above were co-transfected into 293T cells using the Lipofectamine 2000 reagent (Invitrogen, CA, USA) according to the manufacturer’s protocol. After 72 h, the supernatants were harvested and used to infect 5637 and WH cells in the presence of 8 μg/mL polybrene. The stable KLF5 knockdown (KO) 5637 cell line and its control were selected and designated as 5637/KOKLF5 and 5637/KOSC. The pcDNA3.1-based vector and Flag-KLF5 over-expression plasmids used to transfect 5637 cells were described previously [30]. The siRNAs targeting YAP and non-specific control (NC) were purchased from RiboBio (RiboBio Co., Ltd., Guangzhou, China). The specific sequences of the *YAP* gene for siRNA to target were: GCGTAGCCAGTTACCAACA (#1) and CAGTGGCACCTATCACTCT (#2). For mRNA detection, total RNAs were isolated 48 h after transfection. For western blot, cells were harvested 72 h after transfection. For the analysis of cell viability, transfected cells were trypsinized and seeded at 3500–5000 cells per well in a 96-well plate after 24 h.

### 4.6. Real-Time Quantitative PCR (qPCR)

Total RNA from cells was isolated with TRIzol reagent (Life Technologies, Rockville, MD, USA) and reverse-transcribed to cDNA by using the PrimeScript™ RT reagent kit (Takara, Dalian, China). The cDNA was studied using a CFX96 real-time PCR system (Bio-Rad, Hercules, CA, USA) with SYBR Green PCR Master Mix (Takara, Dalian, China) to determine the transcriptional expression of specific genes (seeing Table 1 for primer sequences). GAPDH was used for normalization. Relative gene expression was calculated by the2^−ΔΔ*Ct*^ method.

**Table 1:** Primer sequences used in real-time qPCR.

Gene	Forward Primer	Reverse Primer
*GAPDH*	ATGGGGAAGGTGAAGGTCGG	GACGGTGCCATGGAATTTGC
*KLF5*	CAGAGGACCTGGTCCAGACAAG	GAGGCCAGTTCTCAGGTGAGTG
*WWP1*	ATGTTGTGTGGCATGCAGGA	CTGCAATAGTCGCATTCTTACTTCA
*FBXW7*	CATATGTTGCTCAAAGGTGGCAAG	TGGGACAACAATCCCATATTTGAAG
*SMURF2*	CAAGATTGCTTCAGTTTGTGACAG	GGGCTTTCGGCAGGTTGTT
*YAP1*	AATTTGCCCAGTTATACCTCAGTG	CACATCAAGGCTATGATTCAAACTC
*TAZ*	GCTGAGCGTACAGGCAGACA	AGGAACATAAACCATGGGTCCTT
*CDK6*	TGCACAGTGTCACGAACAGA	ACCTCGGAGAAGCTGAAACA
*AXL*	CGTAACCTCCACCTGGTCTC	TCCCATCGTCTGACAGCA
*ITGB2*	AAGTGACGCTTTACCTGCGAC	AAGCATGGAGTAGGAGAGGTC
*CYR61*	GGTCAAAGTTACCGGGCAGT	GGAGGCATCGAATCCCAGC

### 4.7. Tumor Xenograft Model

Methods to establish a 5637 bladder cancer cell xenograft model were reported previously [31]. Briefly, 1 × 10^6^ 5637 cells in 100 μL of serum-free RPMI-1640 medium mixed with matrigel (1:1 *v*/*v*) were injected subcutaneously into the right flank of 5-week-old male BALB/c nude mice. After 7 days, tumor-bearing mice were randomly divided into vehicle- and curcumin-treated groups (6 mice per group). For curcumin-treated groups, curcumin in phosphate buffered saline (PBS) was injected intraperitoneally at 200 mg/kg/day for 3 weeks. This dosage was proven safe and effective by previous studies in mice [32]. The vehicle group received PBS only. At the end of the experiment, mice was sacrificed, and tumors were weighed and measured to calculate tumor volume (formula: largest diameter × smallest diameter × smallest diameter × 0.5236). Then, tumors were fixed in 4% paraformaldehyde and embedded in paraffin for the next histological examination. Animal care and protocols were approved by the Institutional Animal Care and Use Committee of Xi’an Jiaotong University, and the permit number was SCXK2014-0155 (5 March 2014).

### 4.8. Immunohistochemistry

The EnVisionTM System (DAKO, Carpinteria, CA, USA) was used for IHC staining according to the protocol recommended by the manufacturer. Tumor sections were deparaffinized, rehydrated and subjected to heat-induced antigen retrieval. Endogenous peroxidase and alkaline phosphatase activity were blocked with 3% H_2_O_2_ in methanol for 20 min. The slides were then incubated overnight at 4 °C with primary antibodies: YAP/TAZ (Cell Signaling, #8418, 1:200); KLF5 (Abcam, ab24331, 1:200); PCNA (Santa Cruz, sc-7907, 1:300); cyclin D1 (Santa Cruz, sc-8396, 1:150). After washing three times, slides were incubated with EnVision secondary antibody for 30 min at room temperature. Then, signals were detected by diaminobenzidine (DAB) buffer followed by hematoxylin counterstaining. Slides were viewed and photographed using an Olympus BX51 Microscope (Olympus, Tokyo, Japan).

### 4.9. Statistical Analysis

GraphPad Prism version 5.0 software (GraphPad, San Diego, CA, USA) was used for analyzing differences between two groups (Student’s *t*-test) and performing nonlinear regression analysis. A *p*-value less than 0.05 was considered to be statistically significant.

## 5. Conclusions

Our *in vitro* and *in vivo* results demonstrate that curcumin inhibits the cell growth of bladder cancer at least partially through inhibiting Hippo pathway effectors YAP/TAZ, which induce the accelerated degradation of KLF5 protein and subsequently cause the downregulation of cyclin D1. The study also suggests potential use of curcumin to target the YAP/TAZ/ KLF5/cyclin D1 axis in bladder cancer treatment.

## Supplementary Materials

Supplementary Figures can be accessed at: http://www.mdpi.com/1422-0067/15/9/15173/s1.

## Supplementary Files

Supplementary File 1
