# Supplementary material for: Curcumin Promotes KLF5 Proteasome Degradation through Downregulating YAP/TAZ in Bladder Cancer Cells
Source: Int J Mol Sci. 2014 Aug 28;15(9):15173–87. doi: 10.3390/ijms150915173 (PMC4200832; doi:10.3390/ijms150915173)

Supplementary Information

Figure S1. mRNA expression of KLF5-related E3 ubiquitin ligases WWP1, FBW7 and SMURF2 in 5637 and WH cells after curcumin treatment. Cells were treated with 0, 10 or 20 μM curcumin for 12 h; then, the mRNA expressions of WWP1, FBW7 and SMURF2 were determined by real-time qPCR assay.

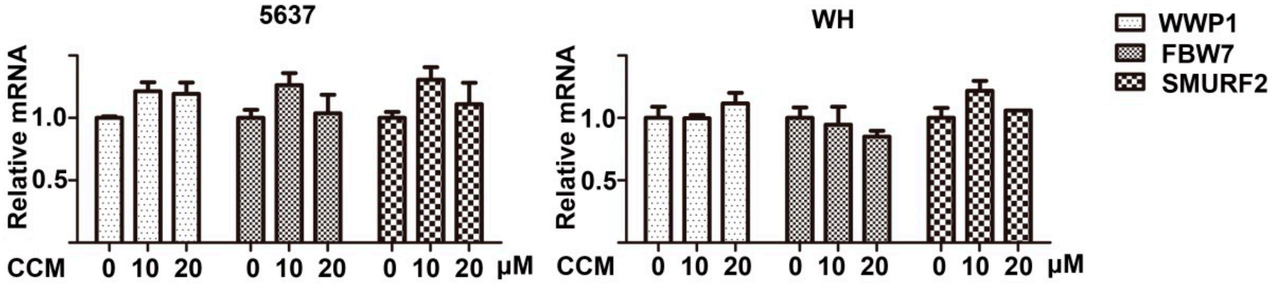

Supplement: Supplementary File 1 [file ijms-15-15173-s001.pdf]
